# A Comparison Effect of Copper Nanoparticles versus Copper Sulphate on Juvenile *Epinephelus coioides*: Growth Parameters, Digestive Enzymes, Body Composition, and Histology as Biomarkers

**DOI:** 10.1155/2015/783021

**Published:** 2015-10-07

**Authors:** Tao Wang, Xiaohua Long, Yongzhou Cheng, Zhaopu Liu, Shaohua Yan

**Affiliations:** Jiangsu Provincial Key Laboratory of Marine Biology, College of Resources and Environmental Sciences, Nanjing Agricultural University, Nanjing 210095, China

## Abstract

Copper nanoparticles (Cu-NPs) are components in numerous commercial products, but little is known about their potential hazard in the marine environments. In this study the effects of Cu-NPs and soluble Cu on juvenile *Epinephelus coioides* were investigated. The fish were exposed in triplicate to control, 20 or 100 *µ*g Cu L^−1^ as either copper sulphate (CuSO_4_) or Cu-NPs for 25 days. The growth performance decreased with increasing CuSO_4_ or Cu-NPs dose, more so in the CuSO_4_ than Cu-NPs treatment. Both forms of Cu exposure inhibited activities of digestive enzymes (protease, amylase, and lipase) found in liver, stomach, and intestine. With an increase in CuSO_4_ and Cu-NPs dose, crude protein and crude lipid decreased, but ash and moisture increased, more so in the CuSO_4_ than Cu-NPs treatment. The Cu-NPs treatment caused pathologies in liver and gills, and the kinds of pathologies were broadly of the same type as with CuSO_4_. With an increase in CuSO_4_ or Cu-NPs dose, the total polyunsaturated fatty acids decreased, but total monounsaturated fatty acids and total saturated fatty acids increased compared to control. Overall, these data showed that Cu-NPs had a similar type of toxic effects as CuSO_4_, but soluble Cu was more toxic than Cu-NPs.

## 1. Introduction

The contamination of aquatic ecosystems by metals is one of the main environmental issues today [1]. In the last decade, the advance of nanotechnology fueled fast growth of the nanotoxicology research and the need for information on potential hazards of this technology in aquatic environments [1, 2].

Copper nanoparticles (Cu-NPs) are one of the most used nanomaterials due to their antibacterial and other properties [3], used for example, in textiles, food storage containers, home appliances, paints, food supplements and so on [4]. Production and use of Cu-NPs likely result in their release into aquatic environments and can lead to unexpected hazards to aquatic organisms [1, 5, 6]. In addition, Cu-NPs could be accumulated in aquatic organisms and transferred to higher trophic levels, representing a health hazard to animals and humans [5, 7].

Currently, concerns have been raised about the effects of Cu-NPs on fish [2, 3, 8] and also about the sublethal effects of NPs on different body systems of fish [8, 9]. However, the majority of the published studies focused on Cu-NPs regarding a lethal dose [8], accumulation [5, 7], stress response [3, 8], osmoregulation [6, 7], and pathology [2]. To our knowledge, the effects of nanometals on digestive enzyme activities, whole-body composition, and fatty acid composition are poorly understood. Furthermore, toxicity thresholds can be rather variable in different species [9–11], and little attention has been given so far to marine teleosts.

*Epinephelus coioides* (grouper), a protogynous hermaphroditic fish, is widely cultured in China and Southeast Asian countries because of its excellent seafood quality and its high market value [12]. Currently,* Epinephelus coioides* species are mainly cultured in floating net cages and earthen ponds in the natural environments that can easily be affected by environment pollution given that discharge of Cu-NPs in the aquatic environment is inevitable [2]. However, as far as we know, there is paucity of data about toxicity of Cu-NPs on* Epinephelus coioides*.

In aquaculture production, the inappropriate management often promotes proliferation of diseases in aquatic organisms [13]. Another problem related to aquaculture is the excessive growth of phytoplankton, particularly blue-green algae [14]. Copper sulphate (CuSO_4_) is employed to control diseases and algae in aquaculture facilities [15]. Copper is an essential micronutrient required for the various functions in biological systems, such as cell structure and enzyme activities of fish [16, 17]. However, excessive Cu in the aquatic environment can be toxic [7, 18]. It has been reported that CuSO_4_ can induce endocrine disruption and change metabolic rates [19, 20], swimming behavior [21, 22], immunological function [2, 8], enzyme activities [8], tissue histology [2, 20], and fatty acid composition [23, 24]. Recently, we found that CuSO_4_ exposure could significantly influence oxidative stress, Na^+^/K^+^-ATPase activity, and cell apoptosis in the liver of juvenile* Epinephelus coioides* [6]. Nevertheless, the physiology effects after* Epinephelus coioides* exposure to CuSO_4_ are poorly understood.

In this study, the main goal was to evaluate the effects of Cu-NPs and CuSO_4_ on growth parameters, activities of the digestive enzymes (protease, amylase, and lipase), whole-body composition, fatty acid composition, and histology of juvenile* Epinephelus coioides*. Results of this study provide an insight into the toxicity mechanisms of Cu-NPs compared with CuSO_4_, aiming to propose margins for a safe use of CuSO_4_ in fish culture.

## 2. Materials and Methods

### 2.1. Experimental Design

Juvenile* Epinephelus coioides* (*n* = 800) were obtained from Shenzhen Dongfang Technology Co., Ltd. China and transferred to indoor tanks for 15 days to acclimate prior to the toxicity assay. During the acclimation period, the juveniles were fed once daily (at 8 am) with commercial feed. After accumulation, selected healthy juveniles (*n* = 525, average weight 3.1 ± 0.2 g) were randomly divided into 15 blue plastic tanks (35 fish per tank, the density approximately 1.0 g fish L^−1^) containing 100 L of simulated seawater. The simulated seawater was made by adding sea salt (purchased from Qingdao Universal Aquaculture Company, China) to the aerated tap water [12].

Three tanks per treatment were randomly allocated and fish were exposed in triplicate to control (no added Cu), 20 *μ*g Cu L^−1^ or 100 *μ*g Cu L^−1^ either as CuSO_4_·5H_2_O or Cu-NPs for 25 days using a semistatic exposure regime (50% water change every 12 h with redosing after each change). The low concentration of 20 *μ*g Cu L^−1^ Cu was selected because it reflects the actual environmental concentration [7]. The high Cu concentration (100 *μ*g Cu L^−1^) was selected because this concentration may be found in some areas with intensive manufacturing industries, agricultural and mining activities, and municipal waste depositions [25]. Water samples were taken before and after each water change and were analyzed for pH, temperature, salinity, dissolved oxygen (tested by YSI 556MPS, USA), total ammonium (by Nessler's reagent spectrophotometry), water hardness (Ca and Mg), and Cu (trace elements were measured by an Inductively Coupled Plasma Optical Emission Spectrometer, ICP-OES, Optima 7000, PerkinElmer, USA). As there were no significant differences (ANOVA, *P* ≤ 0.05) among any tanks in water quality or Cu treatment concentration, data were pooled and were pH 8.1 ± 0.1, water temperature 24 ± 0.5°C, salinity 27.5 ± 0.5 g L^−1^ (w/v), total ammonium 0.15 ± 0.05 mg L^−1^, and total hardness (mg CaCO_3_ L^−1^), 405 ± 4. Continuous aeration was used to ensure dissolved oxygen above 5 mg L^−1^. The photoperiod was 12 h light:12 h dark. The actual concentrations of Cu in the seawater were 2.3 ± 0.1, 21.9 ± 1.4, 102.5 ± 3.1, 20.3 ± 2.8, and 101.2 ± 2.1 *μ*g L^−1^ for the control, 20 and 100 *μ*g Cu L^−1^ as CuSO_4_, and 20 and 100 *μ*g Cu L^−1^ as Cu-NPs treatments, respectively. During the experimental, no fish mortality was observed.

To minimize the influence of hunger throughout the experiment (a requirement in animal husbandry) [17], fish were hand-fed commercial diet (Fish Po, imported from Japan, containing 54% w/w protein and 3.0 ± 0.12 *μ*g Cu g^−1^) twice daily (8 am and 4 pm) at a rate of 2–2.5% w/w fresh body mass each time [12] for 25 days. Feeding was done after each water change, but prior to Cu redosing to minimize the risk of ingestion of Cu-NPs during feeding. Food was eaten within 5 minutes, with no food wasted.

### 2.2. Stock Solutions and Dosing

Stock solutions of CuSO_4_ and suspensions of Cu-NPs were prepared and characterized as described in detail in our previous report [6] using the same stocks. Briefly, powder form of Cu-NPs was purchased from Shanghai Aladdin Co., Ltd. China (manufacturer's information: particles 10–30 nm; purity 99.9%). A fresh 50-mL Cu-NPs stock solution of 1.0 g Cu L^−1^ was prepared at 8 pm daily by dispersing the nanoparticles in ultrapure water (Millipore, ion free and unbuffered), sonicated for 30 min, and stirred for 1 h at room temperature. Primary particle sizes of the Cu-NPs were measured manually from micrographs obtained using transmission electron microscopy (TEM, JEOL JEM-2100, Japan). The primary particle diameters of Cu-NPs in stock suspensions were 85 ± 29 nm (mean ± S.E.M., *n* = 62 particles). According to the method of Sovová et al. [26], the particle size distributions of Cu-NPs in stock suspensions prepared as described above were measured by nanoparticle tracking analysis (NTA, NanoSight LM_10_) in 20 mg L^−1^ dilutions to avoid saturating the instrument. Dilutions of stock suspensions to 20 mg L^−1^ gave sufficient particle tracks (>100 tracks per sample) to provide reproducible data of particle size distribution in the stock suspensions. The stock suspensions were observed to contain a normal distribution of particle sizes, ranging from individual Cu-NPs of 0 to 30 nm to larger particles, almost certainly Cu-NPs aggregates >80 nm. The mean diameter of aggregates in the suspension was 210 ± 130 nm (mean ± S.E.M., *n* = 3).

A stock solution containing 1 g Cu L^−1^ as CuSO_4_ was prepared by dissolving 3.929 g CuSO_4_·5H_2_O in 1 L of ultrapure water (Millipore, ion free and unbuffered). The CuSO_4_ stock solution was used to dose the tanks throughout the 25-day exposure. Dosing of all treatments was carried out following the water change and again the following morning after a subsequent water change.

### 2.3. Fish Sampling

Fish from each tank were weighed at the beginning and end of experiment. Fish were not fed for 24 h before sampling [17]. Six fish were randomly taken from each tank and dissected on an ice tray. The whole liver, stomach, and intestine were removed; fat was cleaned and flushed by normal saline solution (salinity 8.6 g L^−1^, 4°C) and placed into a centrifuge tube (tissues from three out of six fish from each tank were combined) and stored at −70°C for analyzing digestive enzyme activities; the other three fish from each replicate were used for the analysis of fatty acid composition. The remaining fish from each tank were collected for analysis of whole-body composition and for histological observation. For histological observation, the left lobe of liver and the second gill arch on the left were removed and fixed in 10% v/v buffered formal saline (100 mL 40% v/v formaldehyde, 6.5 g NaH_2_PO_4_ (anhydrous), 4 g NaH_2_PO_4_·H_2_O, diluted to 1 L with distilled water, pH 7.2).

### 2.4. Digestive Enzyme Quantification

The total soluble protein content was measured in diluted homogenates by Bradford's method [27] using bovine serum albumin as a standard. Total protease activity was measured according to the method of Gui et al. [28], using casein as substrate with Folin-phenol reagent. Amylase and lipase activities were measured using kits purchased from Nanjing Jiancheng Bioengineering Research Institute of China [29]. The unit of protease activity was defined as the amount of enzyme needed to catalyze the formation of 1 *μ*g of tyrosine per 1 min at 40°C. The unit of amylase activity was defined as 10 mg starch hydrolyzed with the substrate per mg protein in tissue during 30 min at 37°C. The unit of lipase activity was estimated as consumption of 1 *μ*mol substrate (triglyceride) during 1 min at 37°C per g protein in tissue.

### 2.5. Determination of Whole-Body Composition

The fish whole-body samples were freeze-dried and homogenized prior to chemical analysis. Moisture, crude protein, crude fat, and ash contents were determined according to the standard methods [30]. Moisture content was analyzed using a Craft stove at 105°C to a constant weight. Crude protein content was determined by measuring nitrogen (N × 6.25) using the Kjeldahl method. Crude fat content was measured using a micro Soxhlet Foss Soxtec Avanti (Soxtec Avanti 2050 Auto System, Foss Tecator AB, Höganas, Sweden). Ash content was determined by heating the samples in a muffle furnace at 550°C for 12 h.

### 2.6. Determination of Fatty Acids Composition

The lipids from whole-body of juvenile* Epinephelus coioides* were extracted with a chloroform: methanol (2 : 1 v/v) mixture by method of Dubois et al. [31] and esterified with 14% (w/v) boron trifluoride (BF_3_) in methanol according to Yoshioka et al. [32]. The samples were then analyzed using a Shimadzu GC-201 gas chromatograph (Shimadzu Co., Kyoto, Japan) in a cross-linked 5% phenylmethyl silicone gum phase column (30 m × 0.32 mm i.d. × 0.25 mm film thickness; N_2_ as the carrier gas), equipped with flame ionization detection. The injector and detector temperatures were set at 250°C. The column oven temperature was kept at 100°C for 3 min, raised to 180°C at the rate of 10°C min^−1^, and then raised to 240°C at 3°C min^−1^. The relative quantity of each fatty acid present was determined by measuring the area under the chromatograph peak corresponding to that fatty acid.

### 2.7. Histology

Histological analyses followed the standard techniques [33]. Briefly, the samples were dehydrated in rising concentrations of ethanol, cleared in xylene, infiltrated with rising concentrations of liquid paraffin wax at 58°C, and later embedded in paraffin blocks. The sections were cut at 7-*μ*m-thick with a Rotary microtome (MT-1090A, India), and stained using hematoxylin and eosin (H&E). Stained sections were observed by light microscopy (Leica DM750, Switzerland).

### 2.8. Calculations and Statistical Analysis

The relative weight gain rate (WG, %), specific growth rate (SGR, % d^−1^), food conversion ratio (FCR), and protein efficiency ratio (PER) were calculated as follows [28, 34]:

(1) $$\begin{array}{c} \text{WG}\left(\%\right)=\frac{\left[\left(w_{t}-w_{0}\right)\times 100\right]}{w_{0}},\\ {\text{SGR}}_{\text{d}}\left(\%\;\text{da}{\text{y}}^{-1}\right)=\left(\frac{\left(\ln\left(w_{t}\right)-\ln\left(w_{0}\right)\right)}{t}\right)\times 100,\\ \text{FCR}=\frac{C}{\left(w_{t}-w_{0}\right)},\\ \text{PER}=\frac{\left(w_{t}-w_{0}\right)}{\left(C\times \text{protein}\;\;\text{content}\right)}, \end{array}$$

where *w*_*t*_ and *w*_0_ are the final and initial wet body weight (g) of juvenile* Epinephelus coioides*, respectively, *t* is the duration of experiment (25 days), and *C* is the mean total food intake on a dry weight basis [35]. The diet contained 54% w/w protein.

Experimental data were analysed by one-way analysis of variance (ANOVA) using SPSS (18.0; SPSS Inc., Chicago, IL, USA) for Windows. Tukey's test was used to compare differences among treatments. The *P* ≤ 0.05 was considered statistically significant. All data were presented as means ± S.E.M. (standard error of the mean).

## 3. Results

### 3.1. Growth Parameters

With an increase in CuSO_4_ or Cu-NPs concentration, WG, SGR_d_, and PER were decreased compared to control, more so in the CuSO_4_ than Cu-NPs treatments. In contrast, FCR increased with increasing CuSO_4_ or Cu-NPs dose, with the highest FCR at 100 *μ*g Cu L^−1^ as CuSO_4_ (Table 1).

### 3.2. Digestive Enzyme Activities

**Figure 1:**
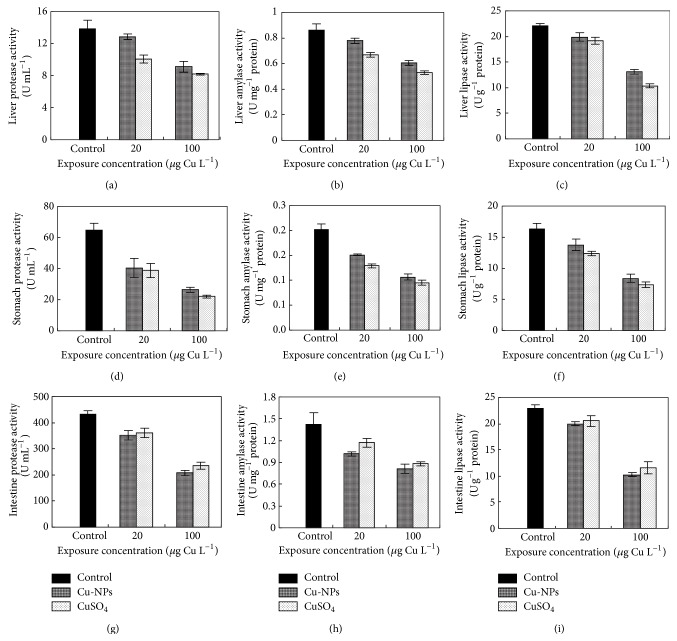
Effects of Cu-NPs and CuSO_4_ on digestive enzyme activities (protease ((a), (d), (g)), amylase ((b), (e), (h)), and lipase ((c), (f), (i)) in liver ((a)–(c)), stomach ((d)–(f)), and intestines ((g)–(i)) of juvenile* Epinephelus coioides* after 25-day exposure.

The activities of protease, amylase, and lipase found in liver, stomach, and intestine were decreased with increasing CuSO_4_ or Cu-NPs dose. For liver and stomach, the CuSO_4_ treatment resulted in lower protease, amylase, and lipase activities than the Cu-NPs treatment, but opposite results were recorded for intestine (Figure 1).

### 3.3. Whole-Body Composition

The whole-body composition was significantly affected by the treatments (Table 2). Crude protein and crude lipid decreased with an increase in CuSO_4_ and Cu-NPs dose, more so in the CuSO_4_ than Cu-NPs treatment. However, ash and moisture increased with an increase in CuSO_4_ and Cu-NPs dose, with the highest ash and moisture at 100 *μ*g Cu L^−1^ as CuSO_4_ (Table 2).

### 3.4. Whole-Body Fatty Acid Composition

As can be seen from Table 3, eicosapentaenoic acid (EPA, C20: 5), docosahexaenoic acid (DHA, C22: 6), and docosapentaenoic acid (DPA, C22: 5) were the lowest at 100 *μ*g Cu L^−1^ as CuSO_4_. The total polyunsaturated fatty acids (∑PUFA) decreased with an increase in CuSO_4_ and Cu-NPs dose, and the lowest ∑PUFA were found at 100 *μ*g Cu L^−1^ as CuSO_4_. However, total monounsaturated fatty acids (∑MUFA) and total saturated fatty acids (∑SFA) increased with an increase in CuSO_4_ and Cu-NPs dose, with the highest ∑MUFA and ∑SFA at 100 *μ*g Cu L^−1^ as CuSO_4_ (Table 3).

### 3.5. Histological Observations

**Figure 2:**
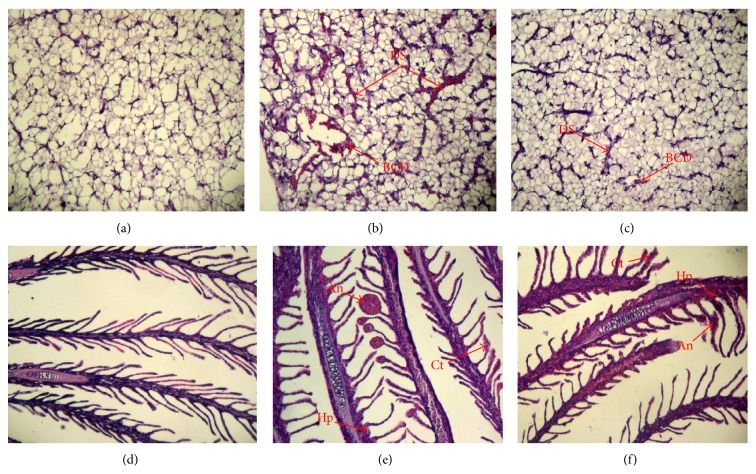
Effects of Cu-NPs and CuSO_4_ on liver and gills morphology of juvenile* Epinephelus coioides* after 25-day exposure. (a) Liver from control, (b) liver from 100 *μ*g Cu L^−1^ as CuSO_4_, (c) liver from 100 *μ*g Cu L^−1^ as Cu-NPs, (d) gills from control, (e) gills from 100 *μ*g Cu L^−1^ as CuSO_4_, and (f) gills from 100 *μ*g Cu L^−1^ as Cu-NPs. In liver, all treatments showed injuries that include blood cell deposition in veins (BCD) and dilatation of sinusoids (DS). In gills, all treatments showed injuries that include hyperplasia (Hp), aneurism (An), and clubbed tips (Ct). Sections were 7-*μ*m thick and stained with haematoxylin and eosin (H&E, ×100).

Liver from control specimens showed the normal structure of sinusoids and vascular system. However, liver from the treatment with 100 *μ*g Cu L^−1^ as either CuSO_4_ or Cu-NPs showed blood cell deposition in veins and dilatation of sinusoids, with the sinusoids becoming irregular in shape (Figure 2). These injuries were greater in the CuSO_4_ than Cu-NPs treatment. No significant histological evidence of injury in liver and gills was observed in the treatment with 20 *μ*g Cu L^−1^ as CuSO_4_ or Cu-NPs (data not shown). In 100 *μ*g Cu L^−1^ as CuSO_4_ or Cu-NPs, the gills showed areas of hyperplasia at the base of the secondary lamellae, clubbed tips at the top of some secondary lamellae, and aneurism in gill filaments (Figure 2).

## 4. Discussion

The water quality of the aquatic environment is the main factor controlling the health of cultured as well as wild fish [36]. Pollution of the aquatic environment by metals is a serious threat to the growth and survival of aquatic organisms including fish [37, 38]. Shaw et al. [7] reported 85% mortality of rainbow trout in 100 *μ*g Cu L^−1^ as CuSO_4_ and 14% mortality in 100 *μ*g Cu L^−1^ as Cu-NPs after 4 days. In our study, almost all parameters evaluated were affected by both forms of Cu exposure, but no mortality was observed after 25 days of exposure to CuSO_4_ or Cu-NPs (up to 100 *μ*g Cu L^−1^). These results may indicate differential sensitivity of different species to Cu toxicity, but further work is necessary to elucidate relevant relationships.

The present study is one of the first reports detailing the effects of Cu-NPs on juvenile* Epinephelus coioides* compared to Cu added as CuSO_4_. Either Cu-NPs or CuSO_4_ exposure decreased WG and SGR_d_ compared to control. Chen et al. [17] reported that the reduction in growth performance was most likely due to two reasons: first, Cu exposure caused increased metabolic expenditure for detoxification and maintenance of homeostasis; second, higher Cu exposure reduced feed intake, which would in turn lead to reduced growth. In our study, the food was eaten within 5 min of presentation, with no waste in any of the treatments, suggesting the increased metabolic expenditure for detoxification and maintenance of homeostasis and/or decreased digestive capabilities were the main reasons for decreased growth performance of juvenile* Epinephelus coioides* rather than decreased food intake. Indeed, growth is a complex phenomenon that partly relies on the digestive capabilities of an organism [39]. Sherwood et al. [40] reported that yellow perch living in lakes subjected to chronic exposure to metals (cadmium, copper and zinc) exhibited greater total energetic costs and lower SGR than fish in reference lakes, despite similar food consumption rates. In the study presented here, diminished growth with either form of Cu exposure was associated with increased FCR and decreased PER. Such results may be disappointing for commercial fish farming due to economic reasons because both Cu-NPs and CuSO_4_ should be strictly monitored in the aquatic environment in actual fish production. This study also found that the CuSO_4_ treatment resulted in a lower growth performance than the Cu-NPs treatment; in addition, the highest FCR and the lowest PER were obtained in the CuSO_4_ treatment, indicating that soluble Cu was more harmful than Cu-NPs to fish growth and food utilization.

During ontogenetic development, marine fish undergo many changes in the structure and function of their digestive system [41, 42]. Digestive enzyme activity (e.g., protease, amylase, and lipase) can be used as an indicator of potential feed utilization and growth differences [39, 43] and to some extent may serve as an indicator of the digestive capacity in relation to the type of feed offered and the properties of aquaculture environments [42, 44]. In our study, the activities of protease, amylase, and lipase found in liver, stomach, and intestine decreased with increasing CuSO_4_ or Cu-NPs dose, suggesting that either Cu-NPs or CuSO_4_ exposure decreased digestive capability of juvenile* Epinephelus coioides*.

Several hypotheses can explain the negative effect of contaminants on digestive enzyme activities: (i) contaminants can act directly on digestive enzymes activities and/or their synthesis [16]; (ii) contaminants can act negatively on the fish behavior, for example, by decreasing the feeding activity, with indirect consequences on digestive enzymes [43, 45]; (iii) the quantity and the quality of available food may also be impacted by the pollution level, leading to a variation in the activities of digestive enzymes [42]. In this experiment, either Cu-NPs or CuSO_4_ exposure had no appreciable effect on feeding activity (as judged from the food being eaten within 5 min of presentation regardless of the treatment). Thus, Cu exposure could act directly on digestive enzyme activities and/or synthesis, which contributed to the lower growth performance.

In liver and stomach, the CuSO_4_ treatment resulted in lower digestive enzyme activities than the Cu-NPs treatment, but the opposite results were recorded for intestine. This result is in accordance with our previous report [6] on Cu accumulation, regarding a negative relationship between Cu accumulation (or Cu exposure concentration) and digestive enzyme activities under either form of Cu exposure. In intestine, the Cu-NPs treatment was associated with higher Cu concentration compared with the CuSO_4_ treatment [6]; hence, digestive enzyme activities were lower in the Cu-NPs than in CuSO_4_ treatment.

Shearer [46] reported that concentrations of crude protein and ash varied during the life cycle and were dependent on the fish size. Abdel-Tawwab et al. [47] assumed that changes in body composition such as crude protein and crude lipid contents could be linked to changes in their synthesis, deposition rate in muscle, and/or differential growth rates. Chen et al. [17] and Shearer [46] reported that deposition of lipids was influenced by several factors, but there was a general trend for percentage body lipids to decrease with decreasing fish size, and any decrease in the percentage of lipids was usually accompanied by an increase in percentage of body water. The results of the present study are in general accord with this. It is likely that the proteins and lipids in fish can be used as energy source for detoxification and the maintenance of homeostasis during metal exposure [48, 49]. In the present study, crude proteins and crude lipids decreased with an increase in CuSO_4_ and Cu-NPs dose, more so in the CuSO_4_ than Cu-NPs treatment, indicating that Cu ions were more harmful to energy stores (such as crude proteins and crude lipids) and weight gain of* Epinephelus coioides* than Cu-NPs. However, Chen et al. [17] found that waterborne cadmium exposure (0.49 and 0.95 mg L^−1^) increased lipid content of yellow catfish. In conclusion, lipid metabolism under metal exposure revealed a complex regulatory mechanism and diverse biological functions of different species and organs [17, 50], so further study is needed.

Metals are highly hazardous xenobiotics. However, optimization of the lipid and fatty acid metabolism may promote the adaptation of an organism to the adverse environmental conditions (cf. Fokina et al. [24]). Sáez et al. [23] reported that the fatty acid composition in the whole body of* Gambusia holbrooki* was obviously changed after the treatment with 0.1, 0.17, or 0.25 mg Cu L^−1^ as CuSO_4_ compared to control. Fokina et al. [24] found that saturated fatty acids (SFA) and monounsaturated fatty acids (MUFA) in gills of mussels decreased significantly after 24 h exposure to 5, 50, or 250 *μ*g soluble Cu L^−1^, but polyunsaturated fatty acids (PUFA) content in gills increased (primarily a rise in EPA, DHA, and AA acids). Borlongan [51] reported that EPA and DHA were the essential fatty acids for fish, but marine fish may have limited ability to synthesize them. Furthermore, DHA and EPA were closely related to the physiological functions of fish, including the antioxidant, immune, and anti-inflammatory responses, as well as protecting the retina and improving vision [12, 52]. In our study, EPA, DHA, and DPA were significantly influenced by the CuSO_4_ or Cu-NPs treatment, with the lowest content at 100 *μ*g Cu L^−1^ as CuSO_4_, indicating that the physiological functions of juvenile* Epinephelus coioides* might be impaired by either form of Cu treatment, even though more so by Cu ions than Cu-NPs. With an increase in CuSO_4_ or Cu-NPs dose, ∑PUFA decreased, but ∑MUFA and ∑SFA increased compared to control. These results may be related to the oxidative stress of juvenile* Epinephelus coioides* under either form of Cu exposure [6].

Metals may alter the structure of cell membranes by stimulating lipid peroxidation [53] caused by free oxygen radicals (superoxide (O_2_^•^) transforming into higher activity oxyradicals, such as hydroxyl (OH^•^) and singlet oxygen (^1^O_2_)). PUFA may be attacked by these oxyradicals, which may stimulate production of lipid radicals and finally aldehydes (such as MDA), ketones, carboxylic acids, and hydrocarbons [24, 54]. In the study presented here, ∑PUFA content decreased and the concentration of MDA increased in juvenile* Epinephelus coioides* (also in our previous study, Wang et al. [6]) compared to control, in accordance to Fokina et al. [24] and D. E. Vance and J. E. Vance [55]. Our study also found the lowest ∑PUFA; in contrast, the highest ∑MUFA and ∑SFA content were found in the treatment with CuSO_4_ (100 *μ*g Cu L^−1^), indicating that fatty acid composition of juvenile* Epinephelus coioides* was affected more strongly by Cu ions than Cu-NPs, and Cu ions were more harmful to the cell membranes than Cu-NPs. Therefore, the degree of Cu toxicity to cells was strongly related to the forms of Cu in the marine environment.

Histological alterations observed in liver and gills were indicative of the fish physiological status, revealing the mechanisms of Cu exposure [56]. Liver and gills were the top two organs for Cu-NPs accumulation in juvenile* Epinephelus coioides* [6]. In the present study, no significant histological evidence of injury was observed in liver and gills in the treatment with 20 *μ*g Cu L^−1^ as CuSO_4_ or Cu-NPs; nevertheless, marine environments with 20 *μ*g Cu L^−1^ pose a risk of Cu accumulation in juvenile* Epinephelus coioides* [6].

The liver is a central compartment of Cu metabolism in fish [2], and it has been used as a reference for analysis of tissue damage caused by environmental pollutants [57, 58]. In the present study, liver from fish exposed to either CuSO_4_ or Cu-NPs (100 *μ*g Cu L^−1^) had blood deposition in veins and dilatation of sinusoids. In other studies, dilatation of sinusoids was one of the most evident signs of liver damage in fish exposed to Cu [8, 10]. Al-Bairuty et al. [2] found that CuSO_4_ (100 *μ*g Cu L^−1^) induced some cellular necrosis and changes in the sinusoid space in liver of rainbow trout; exposure to Cu-NPs (100 *μ*g Cu L^−1^) produced the same type of pathology but affected a greater proportion of liver area and sinusoid space than CuSO_4_. In contrast, in the study presented here, the dilatation of liver sinusoids was greater in the fish treated with 100 *μ*g Cu L^−1^ as CuSO_4_ than Cu-NPs. The difference between the two studies could be due to different fish species and different duration of exposure; it should also be kept in mind that differential susceptibility to Cu could exist between freshwater and seawater fish.

Gills are an important organ for both osmoregulation and respiratory gas exchange, and they were the primary target for toxicity of Cu-NPs [59]. In the present study, gills had areas of hyperplasia at the base of the secondary lamellae, clubbed tips at the top of some secondary lamellae, and aneurism in gill filaments at 100 *μ*g Cu L^−1^ as CuSO_4_ or Cu-NPs. These results were similar with the data recorded by Al-Bairuty et al. [2] and Griffitt et al. [59]. Gomes et al. [3] and Griffitt et al. [59] reported that hyperplasia at the base of the secondary lamellae would increase the diffusion distance for gas exchange, with even a small increase having profound effects on the efficiency of oxygen transfer across gills. Gill injuries from metal exposure were associated with a decrease in arterial oxygen tension, which might be recoverable depending on the extent of the injury (e.g., Zn, Lappivaara et al., [60]). Decreases in arterial oxygen tension in fish exposed to NPs have also been reported [61]. It, therefore, seems probable that the gill injury reported here would cause some hypoxia. Further research on the exercise performance and swimming behaviour of fish exposed to Cu-NPs is required to determine the functional significance of observed gill pathology.

## 5. Conclusions

The present study confirmed that either Cu-NPs or CuSO_4_ exposure had obvious toxicity to juvenile* Epinephelus coioides*. Either form of Cu exposure inhibited digestive enzyme activities, which contributed to the diminished growth performance. The crude proteins and crude lipids in fish might be used as energy source for detoxification and the maintenance of homeostasis during Cu-NPs or CuSO_4_ exposure; thus fish qualities (whole-body composition and fatty acid composition) were obviously affected after either form of Cu exposure. A similar type of pathology was caused by Cu-NPs or Cu metal salts, but greater injuries were found in liver of fish exposed to CuSO_4_ than Cu-NPs. The studied parameters in fish treated by metals could be used as biomarkers, reflecting the adverse effects of marine environment on fish. Finally, considering the importance of this species for fish culture, a further exploration from genomic, transcriptomic, proteomic, and metabolomics should be provided to elaborate for the diminished growth performance after Cu exposure.

## Figures and Tables

**Table 1:** Effect of Cu-NPs and CuSO_4_ on weight gain rate (WG, %), specific growth rate (SGR % d^−1^), food conversion ratio (FCR), and protein efficiency ratio (PER) of juvenile *Epinephelus coioides* after 25-day exposure.

	Control	20 *μ*g Cu L^−1^ as CuSO_4_	20 *μ*g Cu L^−1^ as Cu-NPs	100 *μ*g Cu L^−1^ as CuSO_4_	100 *μ*g Cu L^−1^ as Cu-NPs
WG (%)	105.34 ± 5.16^a^	81.25 ± 5.41^b^	92.19 ± 4.69^b^	47.66 ± 1.97^d^	59.38 ± 2.55^c^
SGR % d^−1^	2.94 ± 0.12^a^	2.40 ± 0.17^b^	2.71 ± 0.13^ab^	1.60 ± 0.06^d^	1.93 ± 0.09^c^
FCR	37.31 ± 1.85^d^	48.51 ± 3.25^c^	42.48 ± 2.16^cd^	82.37 ± 3.26^a^	66.16 ± 2.87^b^
PER	1.61 ± 0.08^a^	1.24 ± 0.08^b^	1.41 ± 0.07^ab^	0.73 ± 0.03^c^	0.91 ± 0.04^c^

Data are means ± S.E.M (*n* = 3). Significant differences (*P* ≤ 0.05) among treatments were indicated by different letters in each row.

**Table 2:** Effect of Cu-NPs and CuSO_4_ on whole-body composition (% on wet weight basis) of juvenile *Epinephelus coioides* after 25-day exposure.

	Control	20 *μ*g Cu L^−1^ as CuSO_4_	20 *μ*g Cu L^−1^ as Cu-NPs	100 *μ*g Cu L^−1^ as CuSO_4_	100 *μ*g Cu L^−1^ as Cu-NPs
Crude protein (%)	16.11 ± 0.89^a^	14.74 ± 0.28^ab^	15.94 ± 0.40^a^	13.53 ± 0.60^b^	14.85 ± 0.29^ab^
Crude lipid (%)	7.94 ± 0.46^a^	6.87 ± 0.28^b^	7.57 ± 0.30^ab^	5.30 ± 0.17^c^	5.83 ± 0.20^c^
Ash (%)	5.03 ± 0.11^b^	5.11 ± 0.14^b^	5.08 ± 0.10^b^	5.83 ± 0.14^a^	5.73 ± 0.17^a^
Moisture (%)	71.32 ± 0.67^d^	73.27 ± 0.30^bc^	72.32 ± 0.33^cd^	75.34 ± 0.43^a^	73.59 ± 0.47^b^

Data are means ± S.E.M (*n* = 3). Significant differences (*P* ≤ 0.05) among treatments were indicated by different letters in each row.

**Table 3:** Effects of Cu-NPs and CuSO_4_ on whole-body fatty acid composition of juvenile *Epinephelus coioides* after 25 days exposure.

Fatty acid	Treatments				
	Control	20 *μ*g Cu L^−1^ as CuSO_4_	20 *μ*g Cu L^−1^ as Cu-NPs	100 *μ*g Cu L^−1^ as CuSO_4_	100 *μ*g Cu L^−1^ as Cu-NPs
	% total fatty acids				
C14:0	1.39 ± 0.06^c^	1.41 ± 0.03^c^	1.33 ± 0.07^c^	2.43 ± 0.07^a^	1.87 ± 0.10^b^
C15:0	0.25 ± 0.03^bc^	0.24 ± 0.02^c^	0.36 ± 0.05^ab^	0.39 ± 0.06^a^	0.28 ± 0.04^abc^
C16:0	18.13 ± 1.31^a^	18.28 ± 1.33^a^	17.69 ± 1.56^a^	20.35 ± 1.44^a^	18.26 ± 1.20^a^
C16:1	2.51 ± 0.07^e^	2.72 ± 0.04^d^	2.96 ± 0.07^c^	4.04 ± 0.09^a^	3.42 ± 0.10^b^
C17:0	0.36 ± 0.08^a^	0.38 ± 0.05^a^	0.37 ± 0.09^a^	0.49 ± 0.11^a^	0.36 ± 0.16^a^
C18:0	8.22 ± 1.22^a^	8.52 ± 1.75^a^	8.57 ± 1.42^a^	8.04 ± 1.11^a^	8.34 ± 1.38^a^
C18:1	17.16 ± 0.06^c^	17.25 ± 0.05^c^	17.09 ± 0.04^c^	19.03 ± 0.07^a^	17.79 ± 0.11^b^
C18:2	21.77 ± 2.03^a^	21.03 ± 2.08^a^	20.45 ± 2.01^a^	21.17 ± 2.33^a^	21.99 ± 1.99^a^
C18:3	1.72 ± 0.04^b^	1.50 ± 0.06^c^	1.72 ± 0.08^b^	1.91 ± 0.09^b^	2.13 ± 0.10^a^
C20:0	0.30 ± 0.03^ab^	0.37 ± 0.03^a^	0.33 ± 0.02^ab^	0.35 ± 0.01^ab^	0.29 ± 0.04^b^
C20:1	0.62 ± 0.07^a^	0.68 ± 0.06^a^	0.72 ± 0.08^a^	0.73 ± 0.04^a^	0.71 ± 0.05^a^
C20:5 EPA	5.48 ± 0.11^b^	5.97 ± 0.18^a^	6.25 ± 0.14^a^	4.00 ± 0.17^c^	5.31 ± 0.12^b^
C22:0	0.10 ± 0.01^c^	0.14 ± 0.09^c^	0.28 ± 0.02^b^	0.47 ± 0.05^a^	0.13 ± 0.01^c^
C22:5DPA	1.65 ± 0.06^ab^	1.51 ± 0.06^b^	1.82 ± 0.09^a^	1.20 ± 0.11^c^	1.59 ± 0.12^ab^
C22:6DHA	17.91 ± 1.02^a^	17.27 ± 1.22^ab^	17.19 ± 1.31^ab^	12.71 ± 1.23^c^	14.54 ± 1.11^bc^
*∑*SFA	28.75 ± 0.39^b^	29.34 ± 0.47^b^	28.91 ± 0.46^b^	32.53 ± 0.41^a^	29.53 ± 0.42^b^
*∑*MUFA	20.29 ± 0.07^d^	20.65 ± 0.05^c^	20.76 ± 0.06^c^	23.80 ± 0.07^a^	21.92 ± 0.09^b^
*∑*PUFA	48.52 ± 0.65^a^	47.29 ± 0.72^ab^	47.42 ± 0.73^ab^	41.00 ± 0.79^c^	45.56 ± 0.69^b^
Others	2.44 ± 0.64^a^	2.72 ± 0.72^a^	2.91 ± 0.72^a^	2.67 ± 0.73^a^	2.88 ± 0.78^a^

Data are means ± S.E.M (*n* = 3). Significant differences (*P* ≤ 0.05) among treatments were indicated by different letters in each row. EPA: eicosapentaenoic acid; DPA: dichloropropanoic acid; DHA: docosahexaenoic acid; *∑*SFA: total saturated fatty acids; *∑*MUFA: total monounsaturated fatty acids; *∑*PUFA: total polyunsaturated fatty acids.
